# Investigation on the Plasma-Induced Emission Properties of Large Area Carbon Nanotube Array Cathodes with Different Morphologies

**DOI:** 10.1007/s11671-010-9784-x

**Published:** 2010-09-28

**Authors:** Qingliang Liao, Zi Qin, Zheng Zhang, Junjie Qi, Yue Zhang, Yunhua Huang, Liang Liu

**Affiliations:** 1 State Key Laboratory for Advanced Metals and Materials, Department of Materials Physics, University of Science and Technology Beijing, 100083 Beijing, China; 2 Department of Physics, Tsinghua-Foxconn Nanotechnology Research Center, Tsinghua University, 100084 Beijing, China

**Keywords:** Carbon nanotubes, Chemical vapor deposition, Plasma-induced emission, High-intensity electron beams

## Abstract

Large area well-aligned carbon nanotube (CNT) arrays with different morphologies were synthesized by using a chemical vapor deposition. The plasma-induced emission properties of CNT array cathodes with different morphologies were investigated. The ratio of CNT height to CNT-to-CNT distance has considerable effects on their plasma-induced emission properties. As the ratio increases, emission currents of CNT array cathodes decrease due to screening effects. Under the pulse electric field of about 6 V/μm, high-intensity electron beams of 170–180 A/cm^2^ were emitted from the surface plasma. The production mechanism of the high-intensity electron beams emitted from the CNT arrays was plasma-induced emission. Moreover, the distribution of the electron beams was in situ characterized by the light emission from the surface plasma.

## Introduction

In the past few years, carbon nanotubes (CNTs) have been extensively investigated due to their remarkable structures and excellent properties [1]. They have also been identified as potential materials for a broad range of useful devices [2,3], especially in the area of field emission devices [4-7]. CNT arrays always have attracted considerable attentions as ideal electron emitters for their excellent field emission properties [5-7]. Many new field emission devices based on CNT arrays were fabricated successfully. In the previous CNT-based devices studies, CNT arrays mainly were applied to the weak current devices under direct current (DC) electric fields. It is well known that plasma-flashover cathodes can generate intense-current electron beams under pulse electric fields and have been used extensively in high-power microwave tubes and accelerators [8-10]. As is mentioned above, CNT arrays have great potentials for the applications of plasma-flashover cathodes due to their excellent field emission properties [5-8]. Whereas the reports that focus on the plasma-induced emission properties of CNT arrays under the high-voltage pulse electric field are very few. Therefore, the studies on the field emission properties of CNTs under the pulse electric field are very important as well as under the DC electric field.

Here, we report the plasma-induced emission characteristics of CNT arrays under the high-voltage pulse electric field. The effects of the ratio of CNT height to CNT-to-CNT distance on the electron emission properties of the CNT arrays were investigated. Moreover, the distribution of electron beams was in situ characterized by light emissions from the plasmas. The production mechanism of the electron beams emitted from the CNT arrays was studied and explained.

## Experimental

Large area CNT arrays have been grown on substrates by a chemical vapor deposition method [5,11], and 2-in. silicon wafers were used as the substrates. Briefly, a 10-nm Al_2_O_3_ layer acting as barrier layer was formed on the substrate surface by evaporation. Then, a 5-nm-thick Fe catalyst layer was e-beam evaporated onto the substrate surface. Finally, the substrates were inserted into the center of a quartz tube furnace. The furnace was heated to about 700°C in the mixed flow of the acetylene and hydrogen. Uniform well-aligned CNT arrays on the 2-in. silicon wafers can be obtained, and the heights of the arrays can be controlled by tuning growth conditions. The height of the as-grown CNT arrays depends on growth time. The growth times of different CNT arrays range from 10 to 80 min. Four kinds of arrays with different CNT heights in the range of 4–16 μm were employed in our experiment. The surface morphologies of the CNT arrays were analyzed by a field emission scanning electron microscopy (SEM). A high-resolution transmission electron microscope (HRTEM) was used to further characterize the synthesized CNTs.

The fabricated samples were placed on copper stages by electrically conductive glue and fixed by copper rings. The CNT arrays were adhere onto the copper stages and assembled into cathodes. Then, the CNT array cathodes were used to next plasma-induced emission tests under the pulse electric field. The high-voltage pulse emission experiments were performed in a diode powered by a pulse-forming network generator at background pressure of 5×10^-4^ Pa [12,13]. The generator has an output double-pulse with about 100-ns duration, and the interval between two pulses was about 400 ns. The anode–cathode gap was 98 mm. During the emission process, the light emission from the CNT array cathode was in situ observed by a charge-coupled device (CCD) camera.

## Results and discussions

**Figure 1:**
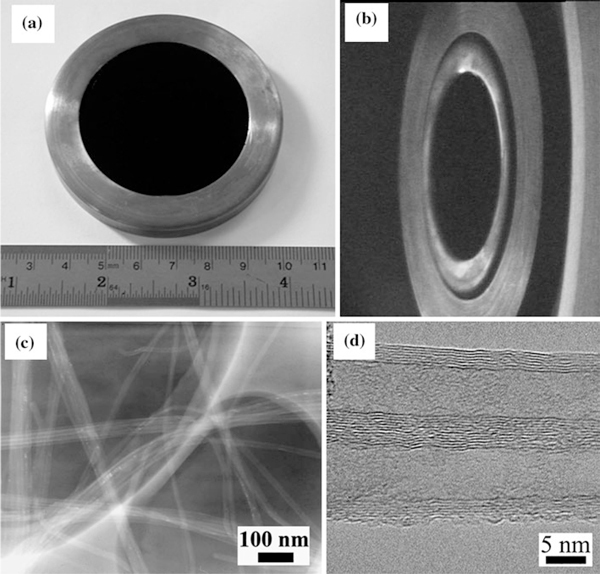
**Morphologies and structures of CNT arrays**: **a** The assembled CNT array cathode on a copper stage, **b** The CCD image of the CNT array cathodes that are not emit, **c** Low-resolution TEM image of the CNTs, **d** High-resolution TEM image of the CNTs.

The assembled CNT array cathode is shown in Figure 1a. The black disk inside the exterior ring is the CNT array film. The emission surface of the array cathode is a disk with 50 mm in diameter. As a whole, the CNT arrays are distribute uniformly on the silicon substrate. Figure 1b shows the CCD image of the cathodes that are not emit, and the middle ellipse of the image is the cathode surface. The only different growth condition among the four kinds of CNT arrays is the growth time, and the CNTs of the four samples have similar diameters. The low-resolution TEM micrograph of the CNTs is shown in Figure 1c, which shows that the CNTs are held together by van der Waals interactions and the CNTs form tight bundles. The diameter of the nanotubes is about 10 nm based on the high-resolution TEM image (shown in the Figure 1d). The high-resolution TEM image reveals that the CNTs are multi-walled. The multi-walled CNTs are relatively clean, and the walls have low defect density.

**Figure 2:**
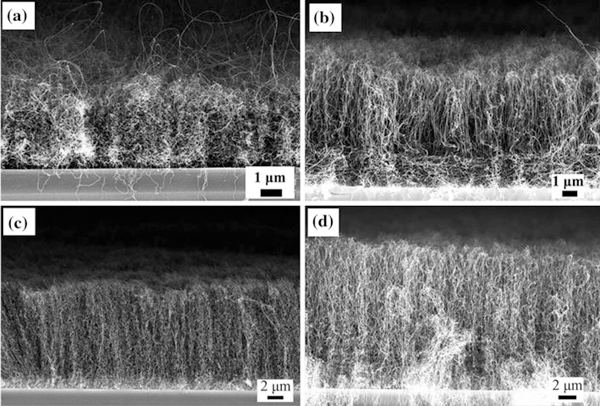
**The side view FESEM images of four kinds of arrays with different CNT heights**: **a** 4 μm, **b** 7 μm, **c** 14 μm, **d** 16 μm.

Figure 2 shows the side view SEM image of four kinds of CNT arrays grown at different growth times. The obvious difference among the four kinds of arrays is the CNT height. The CNT heights of four kinds of arrays are 4, 7, 14 and 16 μm, respectively. The CNTs of the four samples are oriented in a perpendicular fashion and arrange very close with a high density. Besides the heights of the four CNT arrays are different, the CNTs of the four samples have different orientations to the substrates. Most CNTs of the 4-μm height sample are flexural and not perpendicular to the substrate. There are many very long CNTs extruding from the array surface, and the CNTs of the sample are randomly ordered. The CNT arrays of Figure 2a have the shortest growth time among the four kinds of arrays. During the short growth process, the growth temperature rising and falling rapidly and the CNTs have different growth velocity. A lot of CNTs grow at unstable high velocity during the short grown process, but most grown slowly and uniformly. Therefore, there are a lot of long CNTs appeared in the Figure 2a, and the arrays lose the uniformity. If the growth time is long enough, the growth of arrays would reaches a steady state and the arrays would grow very uniform. The CNT arrays of the 7-μm height sample arranges more orderly than that of the 4-μm height sample. A few nanotubes of the 7-μm height sample are flexural at the root. The distributions of the CNTs become regular with the height of the CNT arrays increasing. The CNTs of the 14- and 16-μm height samples are more uniform than that of the 4- and 7-μm height samples. The CNTs have uniform diameters and heights, and they are aligned regularly one by one. CNT arrays with four different heights in the range of 4–16 μm have been fabricated. The nanotubes of the four kinds of arrays have different CNT heights and similar CNT densities. Therefore, the four samples have different ratios of CNT height to CNT-to-CNT distance. The intertube distance is about 130~150 nm, and the ratios of CNT height to CNT-to-CNT distance of four kinds of arrays are 31, 54, 108 and 123, respectively. As the growth times increase, the heights of the CNT arrays increase and the ratios of CNT height to CNT-to-CNT distance increase simultaneously.

**Figure 3:**
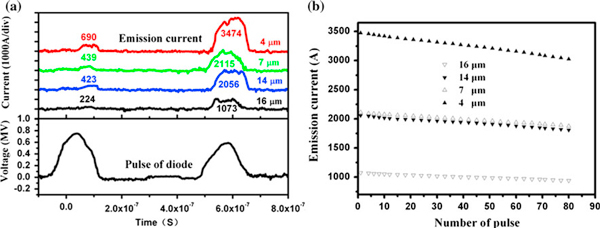
**a The waveforms of diode voltage and emission currents from the four kinds of array cathodes with different CNT heights**. **b** Dependence of the cathode current on the number of pulses.

An application of the pulse electric field caused the appearance of an intense current electron emission from the CNT array cathodes. Figure 3 gives the emission current waveforms and the emission stability curves from the four kinds of array cathodes with different CNT heights. During one double-pulse, the highest voltages of two pulses are about 0.75 and 0.58 MV, respectively. Under the same diode voltage, the emission current waveforms of four kinds of array cathodes have same characteristics. The emission currents have big differences between the first pulse and the second pulse. The big difference of emission currents attributes to the formation and expansion of the surface plasma [8-10]. For the four kinds of arrays with different CNT heights of 4, 7, 14 and 16 μm, the highest emission currents are 3474, 2115, 2056 and 1073 A, respectively. The CNT arrays of four kinds of cathodes have different ratios of CNT height to CNT-to-CNT distance. The difference in the emission currents of the CNT arrays can be caused by only the ratios of CNT height to CNT-to-CNT distance. With increase in the ratios of CNT height to CNT-to-CNT distance, the emission currents decrease gradually. The 4-μm height CNT array has the highest emission current among the four kinds of CNT arrays. The average electric field of the second pulse is about 6 V/μm, and the corresponding highest emission current density of 4-μm height CNT array is about 170–180 A/cm^2^. The relationship between the emission currents and the number of pulses for the four kinds of arrays is presented in Figure 3b. Along with the continuance of the pulse emission, the CNT arrays would lose the emission ability gradually [13-15]. The results show that after 80 pulses, the emission currents of the four kinds of CNT array have about from 11.3 to 12.9% reductions.

**Figure 4:**
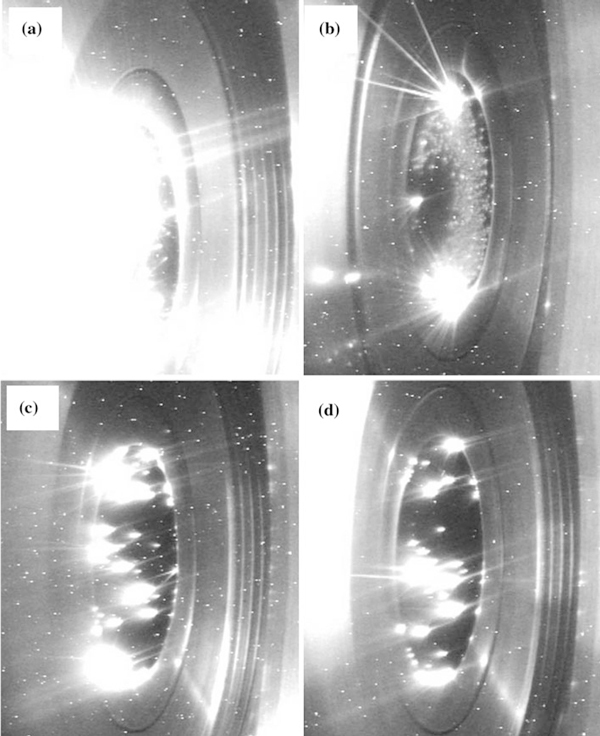
**The CCD images of four kinds of CNT array cathodes that are emitting**: **a** 4 μm, **b** 7 μm, **c** 14 μm, **d** 16 μm.

Figure 4 shows the CCD images of the four kinds of CNT array cathodes that are emitting. Compared with the CCD image of the cathodes that are not emit (shown in Figure 1b), it can be found that the CCD images of Figure 4 have bright light. The bright light captured by the CCD camera was considered as the light emission from plasmas on the cathode surface [8-10]. The luminescent zones on the cathode surface are emission sites. The CCD images show the spatial distribution of the emission sites and the plasmas. The distribution of the emission sites on the CNT arrays is very similar to that of the coated CNT cathode [12,13]. Figure 4a is the CCD image of the 4-μm height cathode. The luminescence on the cathode surface is very intense and uniform. The CCD image shows that the emission current is very intense and almost whole cathode surface can emit electrons. The luminescence of the 7-μm height cathode becomes weak obviously relative to the 4-μm height cathode, as shown in Figure 4b. It can be seen that many separate emission sites distribute on the cathode surface. The brightness and area of the luminescence are less than these of the 4-μm height cathode. It has been known that the 4-μm height CNT arrays have the higher emission current. Therefore, the CCD images can reflect the intensity of emission currents. The CCD images of the 14- and 16-μm height cathodes are shown in Figure 4c and Figure 4d, respectively. The brightness and area of luminescence reduce in contrast with that of the previous short CNT array cathodes. The emission area of the 14-μm height cathode is larger than that of the 16-μm height cathode. The emission current is in direct proportion with the brightness and area of light emission from the plasma. The CCD images reconfirmed that the emission currents of the CNT array cathodes decrease with the increase in the ratios of CNT height to CNT-to-CNT distance.

Many studies were carried out on the influential factors on the electron emission properties of CNT arrays [16-19]. For high-density nanotube arrays, field-screening effects of neighboring tubes reduce the field enhancement, and thus the emission current decreases. When the nanotube height is longer than the intertube distance, the field emission is decreased with the increase in the nanotube height [17-19]. All the CNT arrays in this study have very high densities, and the field-screening effects become the dominant disadvantageous factor to the electron emission. Therefore, the screening effects of the short CNT arrays are less than that of these long CNT arrays. The short CNT arrays have better emission properties than these long CNT arrays. With the increase in the ratio of CNT height to CNT-to-CNT distance, the emission currents of CNT arrays decrease reversely due to the screening effects. Moreover, the plasma forms on the cathode surface and influences the emission currents of cathodes. The effect cathode radius can be calculated by Child-Langmuir Law [20,21]. A series of results on the plasma-induced emission properties of the CNT arrays are shown in Table 1. Along with the increase in CNT array height, the effect cathode radiuses and emission areas decrease. Based on the above analysis, the areas of plasma layer decrease with the increase in the array height. Therefore, the emission area is proportional to the plasma area. The plasma layer is beneficial to the increase in emission currents. The results show that the screening effects are diminished due to the presence of a plasma layer.

**Table 1:** Typical results of plasma-induced emission from the ZnO nanorod array cathode

Samples	Pulse	*I* (A)	***P***_**i **_**(perv)**	***d***_**eff **_**(cm)**	***r***_**eff **_**(cm)**
4 μm	1	690	1.06 × 10^-6^	9.80	3.73
	2	3474	8.75 × 10^-6^	3.41	
7 μm	1	439	6.75 × 10^-7^	9.80	2.98
	2	2115	5.33 × 10^-6^	3.49	
14 μm	1	423	5.91 × 10^-7^	9.80	2.78
	2	2056	3.51 × 10^-6^	4.00	
16 μm	1	223	3.11 × 10^-7^	9.80	2.02
	2	1073	1.50 × 10^-6^	4.46	

*I*_c_ is the cathode current, *P*_i_ is the diode perveance, *d*_eff_ is the effective diode gap and *r*_eff_ is the cathode radius

**Figure 5:**
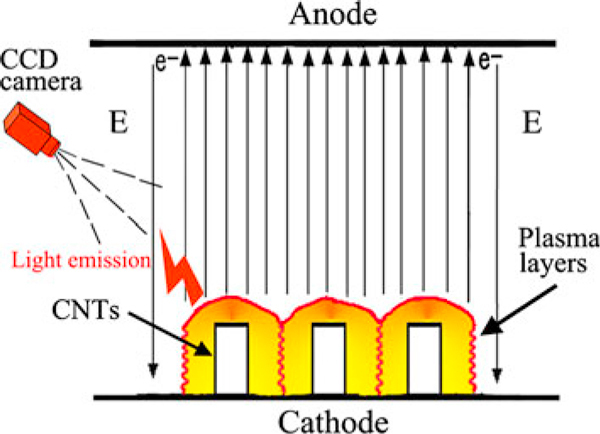
**The electron emission model of CNT arrays under the high-voltage pulse electric field**.

A tube configuration of CNTs enables them to absorb gas, and the dense CNTs can adsorb a large amount of gas molecules [22,23]. The CNTs can emit high-intensity electron beams under the high-voltage pulse electric field. Under the effect of the high-intensity electron beams, the adsorbent gas molecules are easy to become ionization [8-10,23]. The CCD camera has captured the light emission from the CNT arrays. This demonstrates that plasmas formed on the array surface during the emission process. The electron emissions of CNT arrays under the pulse electric field are not pure field emission. The production mechanism of the high-intensity electron beams from the CNT arrays is plasma-induced emission. The emission model of the CNT arrays under the high-voltage pulse electric field is shown in Figure 5. Above all, the plasma layer forms on the cathode surface under the effect of high-intensity electron beams. Subsequently, the cathode surface is covered by plasma, and the electron beams are extracted from the surface plasma. The results demonstrate that the CNT arrays have the ability of emitting high-intensity electron beams under the pulse electric field. The CNT array cathodes are expected to be applied to high-power vacuum electronic devices as electron beam sources.

## Conclusions

In this study, large area well-aligned CNT arrays with different morphologies were fabricated. The plasma-induced emission properties of the CNT arrays with different CNT heights under the pulse electric field have been investigated. The ratios of CNT height to CNT-to-CNT distance have considerable effects on their electron emission properties. As the ratios increase, the emission currents of the CNT arrays decrease due to the screening effects. Plasmas formed on the array surface during the emission process, and high-intensity electron beams of about 170–180 A/cm^2^ were obtained from the CNT arrays. CNT arrays are excellent candidate as intense-current electron beam sources and can be applied to high-power vacuum electronic devices in the near future.
